# Structural and Functional Characterization of the FadR Regulatory Protein from *Vibrio alginolyticus*

**DOI:** 10.3389/fcimb.2017.00513

**Published:** 2017-12-12

**Authors:** Rongsui Gao, Defeng Li, Yuan Lin, Jingxia Lin, Xiaoyun Xia, Hui Wang, Lijun Bi, Jun Zhu, Bachar Hassan, Shihua Wang, Youjun Feng

**Affiliations:** ^1^Department of Medical Microbiology and Parasitology, Zhejiang University School of Medicine, Hangzhou, China; ^2^Institute of Biophysics, Chinese Academy of Sciences, Beijing, China; ^3^School of Life Sciences, Fujian Agriculture and Forestry University, Fuzhou, China; ^4^Department of Microbiology, Nanjing Agricultural University, Nanjing, China; ^5^Department of Microbiology, Perelman School of Medicine, University of Pennsylvania, Philadelphia, PA, United States; ^6^Department of Biochemistry and Biophysics, University of North Carolina at Chapel Hill, Chapel Hill, NC, United States

**Keywords:** FadR, ligand, fatty acid sensing, virulence, *Vibrio alginolyticus*

## Abstract

The structure of *Vibrio cholerae* FadR (VcFadR) complexed with the ligand oleoyl-CoA suggests an additional ligand-binding site. However, the fatty acid metabolism and its regulation is poorly addressed in *Vibrio alginolyticus*, a species closely-related to *V. cholerae*. Here, we show crystal structures of *V. alginolyticus* FadR (ValFadR) alone and its complex with the palmitoyl-CoA, a long-chain fatty acyl ligand different from the oleoyl-CoA occupied by VcFadR. Structural comparison indicates that both VcFadR and ValFadR consistently have an additional ligand-binding site (called site 2), which leads to more dramatic conformational-change of DNA-binding domain than that of the *E. coli* FadR (EcFadR). Isothermal titration calorimetry (ITC) analyses defines that the ligand-binding pattern of ValFadR (2:1) is distinct from that of EcFadR (1:1). Together with surface plasmon resonance (SPR), electrophoresis mobility shift assay (EMSA) demonstrates that ValFadR binds *fabA*, an important gene of unsaturated fatty acid (UFA) synthesis. The removal of *fadR* from *V. cholerae* attenuates *fabA* transcription and results in the unbalance of UFA/SFA incorporated into membrane phospholipids. Genetic complementation of the mutant version of *fadR* (Δ42, 136-177) lacking site 2 cannot restore the defective phenotypes of Δ*fadR* while the wild-type *fadR* gene and addition of exogenous oleate can restore them. Mice experiments reveals that VcFadR and its site 2 have roles in bacterial colonizing. Together, the results might represent an additional example that illustrates the *Vibrio* FadR-mediated lipid regulation and its role in pathogenesis.

## Introduction

The *Vibrionaceae* is a family of Gram-negative bacteria inhabiting fresh/salt water environments that comprised a collection of pathogenic species (Thompson et al., 2005). *V. cholerae* is the notorious cause of cholera transmitted by contaminated water. *V. parahaemolyticus* is a zoonotic pathogen that cause serious food-borne infections from poorly-cooked sea-food (e.g., gastroenteritis and septicemia). Both *V. vulnificus* and *V. alginolyticus* are opportunistic bacterial pathogens since they cause severe wound infection and even fatal septicemia. The two major virulence determinants of *Vibrio cholerae* referred to toxin-coregulated pilus (TCP) (Taylor et al., 1987) and cholera toxin (CT) (Matson et al., 2007), which are coordinated by two independent activators ToxR, a transmembrane regulatory protein (Miller et al., 1987), and ToxT, a member of AraC family transcription factors (Champion et al., 1997). It seems true that the regulated expression of fatty acid metabolism is implicated into *Vibrio* pathogenicity in that (i) inactivation of *fadR* impairs virulence of *V. vulnificus* in mice (Brown and Gulig, 2008); (ii) the disruption of *fadD* gene encoding a long-chain fatty acyl coenzyme A ligase affected production of virulence factors, CtxAB and TcpA (Ray et al., 2011); and (iii) unsaturated fatty acids from bile interfered with bacterial mobility via remodeling bacterial membrane structure (Giles et al., 2011) and inhibited the expression of virulence factors (CtxAB and TcpA) by decreased DNA-binding activity of ToxT in *V. cholerae* (Brown and Gulig, 2008; Giles et al., 2011; Yang et al., 2013; Kovacikova et al., 2017). More recently, Kovacikova et al. (2017) proposed that the *V. cholerae* FadR controls the expression of the virulence cascade by indirect-regulation of ToxT via two different mechanisms (Kovacikova et al., 2017). In contrast to the *E. coli* FadR paradigm, its *Vibrio* homologs with an insert of 40 aa exhibit stronger ability of binding LC acyl-CoA thioesters (Heidelberg et al., 2000; Iram and Cronan, 2005). The structural mechanism of *V. cholerae* FadR relies on an additional acyl-CoA ligand binding site formed mainly by the 40-residue insert (Shi et al., 2015).

The paradigm *E. coli* FadR regulator, belonging to a GntR-type transcription factor, is a dimeric protein that is composed of an N-terminal DNA-binding motif and a C-terminal ligand-interaction domain (van Aalten et al., 2000, 2001; Xu et al., 2001). The *E. coli* FadR acts as a moderate global regulator in that it negatively regulates more than 12 *fad* genes [such as *fadBA* (Iram and Cronan, 2006), *fadM* (Feng and Cronan, 2009a), *fadH* (Feng and Cronan, 2010), etc.] and it positively regulates, the two key genes (*fabA* and *fabB*) of UFA synthesis (Henry and Cronan, 1992; Campbell and Cronan, 2001). In addition, FadR also activates the *fabHDG* operon required for SFA synthesis in *E. coli* (My et al., 2013). The physiological ligand of FadR is LC fatty acyl-CoA thioesters. Upon FadR binding of these ligands, FadR is released from all its DNA-binding sites (Henry and Cronan, 1992; Cronan, 1997). This mechanism explains the fatty acid-inducible expression of *fad* regulon (Overath et al., 1969; Klein et al., 1971) plus the fatty acids-mediated repression of *fabA*/*fabB* expression (Henry and Cronan, 1991; Campbell and Cronan, 2001). Although the structural basis for *E. coli* FadR regulation is well understood from the x-ray crystal structures of *E. coli* FadR alone (van Aalten et al., 2000), bound DNA (van Aalten et al., 2001; Xu et al., 2001) and in a complex with its ligand myristoyl-CoA (van Aalten et al., 2001), further investigations unexpectedly suggested that FadR orthologs display appreciable functional diversity (Iram and Cronan, 2005) in that FadR proteins from diverse y-proteobacteria showed large differences in the ability to regulate *E. coli fad* regulon.

**Figure 1:**
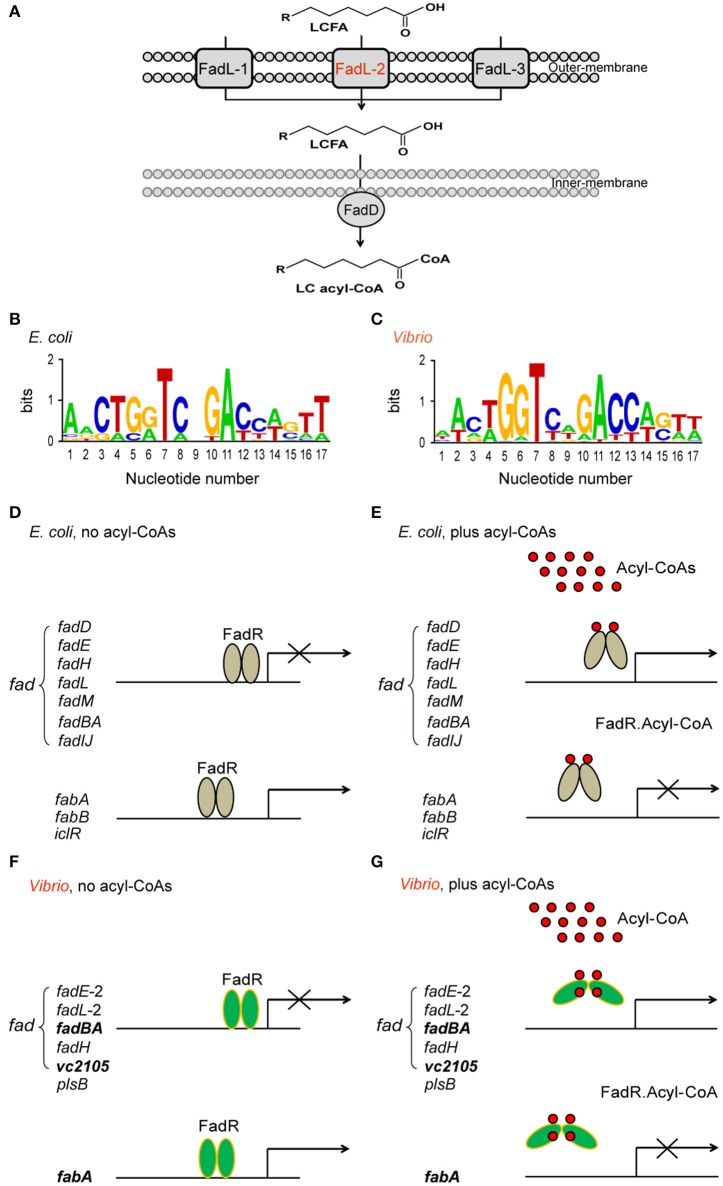
Working model for *Vibrio* FadR-mediated fatty acid sensing and regulation. **(A)** Schematic representative for uptake and activation of long-chain fatty acids by FadL-FadD system of *V. cholerae* N16961. The long-chain fatty acids are transported by FadL membrane protein from the environment into periplasm and then activated by inner-membrane associated protein FadD to give LC acyl-CoA in cytosol. Among three putative FadR homologs encoded by *V. cholerae* N16961 genome, FadL2 (in red) is only one gene with a predicted FadR-binding site. LCFA is an abbreviation of long chain fatty acid. **(B)** Sequence logo for FadR-specific palindromes from *E. coli* MG1655. **(C)** Sequence logo for FadR-specific palindromes from *Vibrio* genus. The sequences of the known *E. coli* FadR sites were sampled from *E. coli* K-12 MG1655 (http://regprecise.lbl.gov/RegPrecise/regulon.jsp?regulon_id=10286), and the putative *Vibrio* FadR sites were collected from 9 *Vibrio* species (http://regprecise.lbl.gov/RegPrecise/sites.jsp?regulog_id=1762) (Novichkov et al., 2013). **(D)** *E. coli* FadR represses *fad* expression, but activates transcription of *fabA*/*B* and *iclR* on the condition without acyl-CoA thioesters. **(E)** Presence of long chain acyl-CoAs releases *E. coli* FadR protein from its cognate targets, resulting in an induction of *fad* expression and transcriptional inactivation of *fabA*/*B* and *iclR*. **(F)** *Vibrio* FadR represses the expression of limited *fad* members, whereas only activates *fabA* transcription on the condition with poor acyl-CoA species. **(G)** The generated long chain acyl-CoAs releases *Vibrio* FadR protein from its cognate targets, which consequently induces the expression of limited *fad* members, whereas only inactivates *fabA* transcription. The three representative genes (*vc2105, fadBA*, and *fabA*) are highlighted in bold and italic letters, whose ability of binding the *Vibrio* FadR regulator is demonstrated with EMSA (and/or SPR). The gray oval denotes *E. coli* FadR regulatory protein whereas the green oval denotes *Vibrio* FadR regulator. The small red circle represents the acyl-CoA pool. *E. coli* FadR has only one ligand-binding site in each monomer, whereas *Vibrio* FadR has two ligand-binding sites in each monomer.

In views of genomic context of lipid metabolism (Figure 1), the *Vibrio* species, like *V. cholerae*, seem quite different from *E. coli* (and *S. enterica*) because they have two chromosomes that together encode all the enzymes of fatty acid metabolism. Unlike the paradigm “FadL-FadD” fatty acid transport system in *E. coli, Vibrio* encodes three *fadL* orthologs only one of which has the FadR-binding site recognized by *Vibrio* (Figure 1A) (Heidelberg et al., 2000). This might facilitate more efficient scavenging low level of fatty acids from the dilute marine environment (van den Berg et al., 2004). Relative to *E. coli* which has 12 FadR-regulated genes (Figures 1B–E), the predicted FadR-binding palindrome is detected in only 7 *Vibrio* genes including 5 known *fad*/*fab* genes, suggesting contraction of *fad* regulon (Figures 1F,G) (Zhang et al., 2015; Gao et al., 2016). Of note, the *Vibrio plsB* gene is under FadR control (Figures 1F,G), whereas the *E. coli* counterpart has no effect on this gene (Feng and Cronan, 2011). In contrast to the FadR of *E. coli* and *S. enterica*, all the three *Vibrio* FadR proteins (ValFadR for *V. alginolyticus*, VcFadR for *V. cholerae* and VpFadR for *V. parahaemolyticus*) consistently have an unusual insert of 40 residues (Figure S1), one of which had already been demonstrated to constitute an additional ligand-binding site in the case of VcFadR (Shi et al., 2015). It explains at least (if not all) why *V. cholerae* has potent ability in fatty acid sensing.

Given that knowledge of fatty acid metabolism in *V. alginolyticus* is limited, here we report structural and functional characterization of *V. alginolyticus* FadR. The snapshot of the ValFadR-ligand complex structure also illustrates an additional ligand-binding site. Mice infection experiments suggest that both FadR and the second ligand-binding site might be required for bacterial successful colonization. Together, these results constitute an additional example facilitating us to further understand *Vibrio* fatty acid signaling by FadR and its relevance to bacterial virulence. It is rational that development of small molecule inhibitors targeting the FadR-mediated fatty acid sensing might be a promising strategy against *Vibrio* infections.

## Results

### Characterization of the *V. alginolyticus* FadR

**Figure 2:**
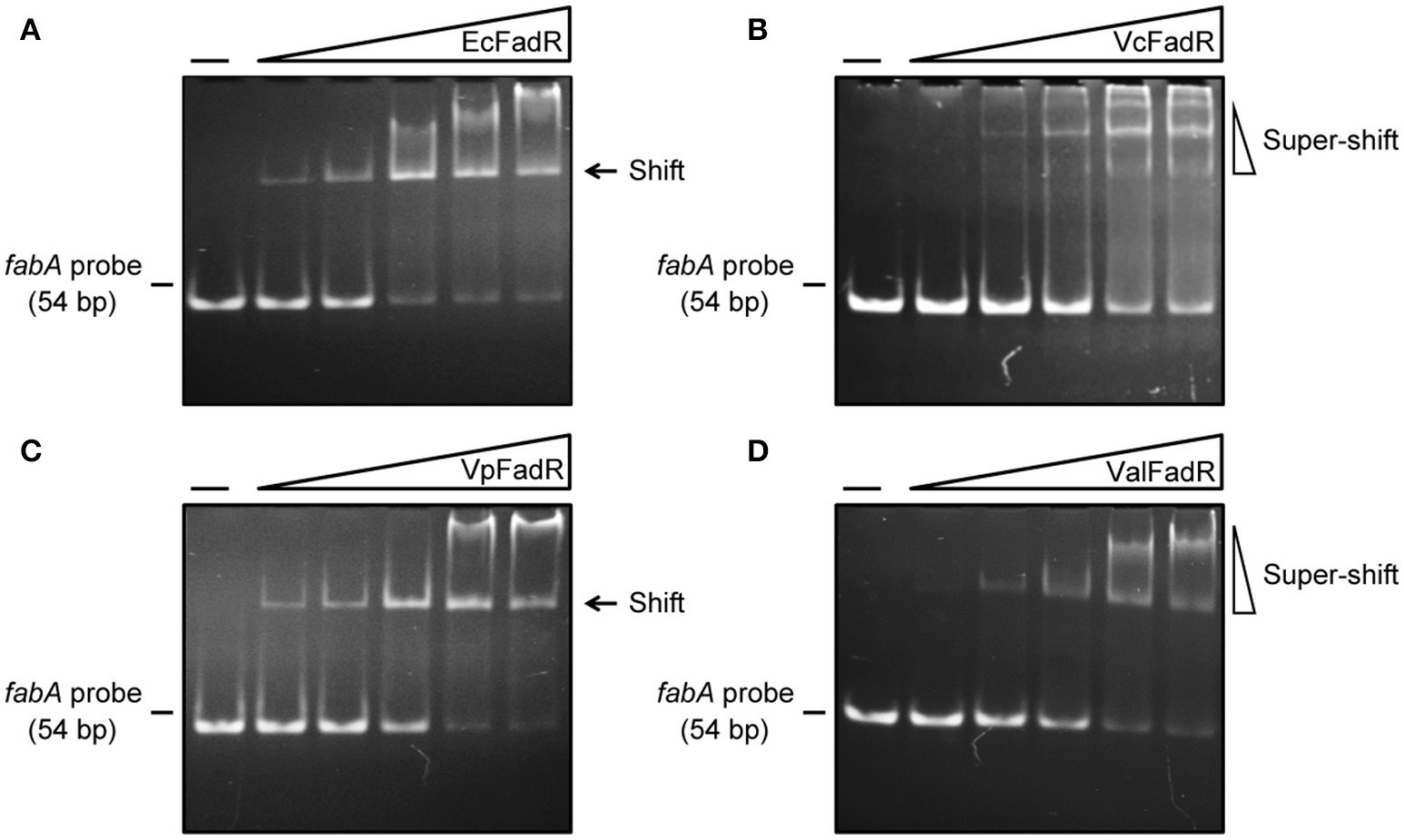
Binding of *fabA* to four types of FadR regulatory proteins. **(A)** Gel filtration analyses for interaction of EcFadR with *fabA*. **(B)** Use of EMSA assays to detect interplay between *fabA* and VcFadR. **(C)** EMSA-based analyses for interaction of *fabA* with VpFadR. **(D)** Physical evidence for binding of ValFadR to *fabA*. The gel shift experiments were carried out using 9% native PAGE, and a representative result from no less than three independent experiments is given. In each assay (15 μl in total), the *fabA* probe (5 pmol) is supplemented, and varied level of FadR protein is indicated in the right hand five lanes of each panel (left to right) (2, 5, 10, 15, and 20 pmol). The four types of FadR proteins referred to EcFadR, VcFadR, VpFadR and ValFadR, respectively. The minus sign denotes the absence of FadR protein. FadR protein at high level precipitates easily in the wells of gel or triggers super-shift in the gel shift experiments.

**Figure 3:**
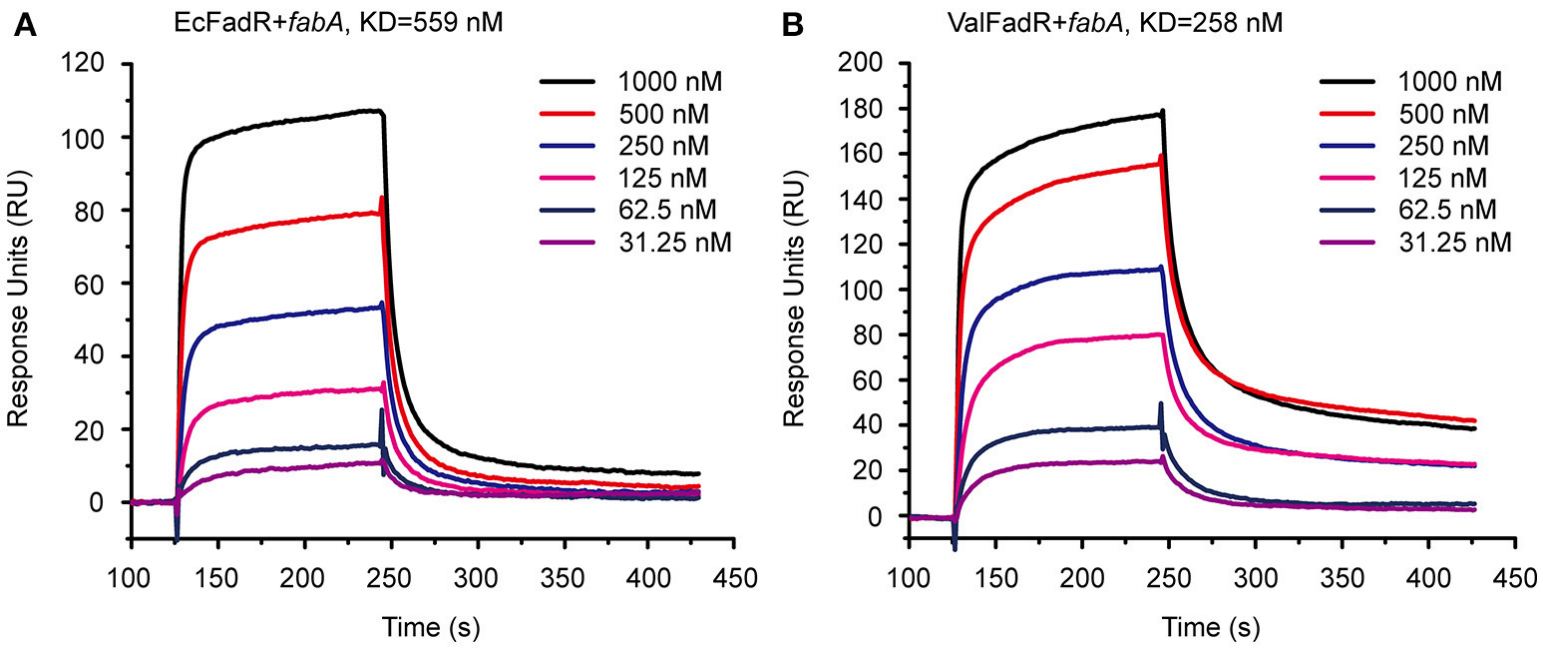
SPR-based assays for binding of *fabA* to the FadR regulator. **(A)** SPR analysis of the *E. coli* FadR binding to the *fabA* promoter. **(B)** SPR assay for the *V. alginolyticus* FadR binding to the *fabA* promoter. SPR assays were conducted to study the affinity and kinetics of FadR proteins and a representative result is given regarding to EcFadR and ValFadR. FadR protein at various concentrations (typically 31.25–1,000 nM) were injected over the immobilized DNA probe covering the FadR palindrome of *fabA* gene. The immobilization level of *fabA* is 34 RU. KD, kd/ka, ka, association constant; kd, disassociation constant; RU, response units.

We are interested in probing if similar scenarios occur in *V. alginolyticus*. Thus, four types of FadR proteins (namely ValFadR, VcFadR, VpFadR, and EcFadR), were over-expressed and purified with nickel-affinity column, whose purity was judged by SDS-PAGE (Figure S2C). Size exclusion analyses confirmed that all the *three Vibrio* FadR proteins (ValFadR and VpFadR, in Figure S2A; VcFadR in Figure S2B) can form dimeric structure in solution, which is almost identical to that of EcFadR (Figure S2A). Together with surface plasmon resonance (SPR)-based detection, EMSA experiments were also applied to address binding abilities of the putative *Vibrio* FadR palindromes (Figures 1C,F,G). A 54 bp of DNA fragment covering the FadR palindrome in the upstream of the *Vibrio fabA* gene is designated herein as *fabA* probe. In general agreement with that of EcFadR (Figure 2A), gel shift assays also confirmed that the *fabA* probe interacts efficiently with ValFadR (Figure 2D). Similar scenarios were also seen with the other two *Vibrio* FadR regulatory proteins VcFadR (Figure 2B) and VpFadR (Figure 2C). Of note, super-shift bands appeared in the cases of VcFadR (Figure 2B) and ValFadR (Figure 2D). In addition, surface plasmon resonance (SPR) results showed clearly that the *fabA* probe binds to both EcFadR (Figure 3A) and ValFadR (Figure 3B) in the dose-dependent manner. The binding affinity of *fabA* to EcFadR and ValFadR is estimated to be 5.59 × 10^−7^ M (Figure 3A) and 2.58 × 10^−7^ M (Figure 3B), respectively. Indeed, we recently extended *fad* regulon to a newly-identified auxiliary *fad* member, *vc2105* of *V. cholerae* (equivalent to *vp0834* of *V. parahaemolyticus*) (Novichkov et al., 2013; Gao et al., 2016). Therefore, we believed that ValFadR is functional and its regulatory role is relatively-conserved (Figure 1).

### Distinct ligand-binding manner of ValFadR and EcFadR

**Figure 4:**
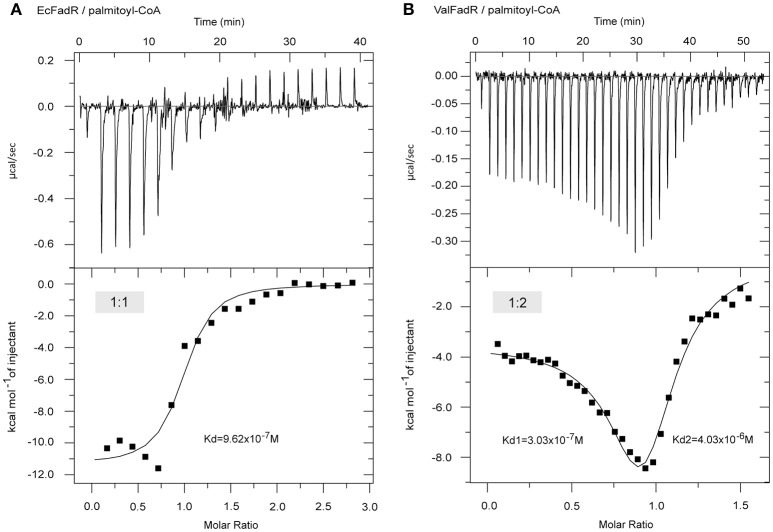
ITC-based evidence for distinct binding pattern of the palmitoyl-CoA ligand to FadR regulatory protein from *E. coli* and *Vibrio alginolyticus*. **(A)** ITC profile of the *E. coli* FadR binding its ligand palmitoyl-CoA. **(B)** The use of ITC to assay interplay between the *V. alginolyticus* FadR and its ligand palmitoyl-CoA. Ec, *E. coli*; Val, *V. alginolyticus*.

Consistent with earlier observations with EcFadR (Feng and Cronan, 2010, 2012) and VcFadR (Feng and Cronan, 2011), *in vitro* gel shift assays illustrated that only Long-chain fatty acyl-CoA species [e.g., palmitoyl-CoA (C16:0), palmitoleyl-CoA (C16:1), stearoyl-CoA (C18:0), and oleoyl-CoA (C18:1), in Figure S3] release the ValFadR protein from its cognate DNA, whereas the medium-chain fatty acyl-CoA thioesters [such as nonanoyl-CoA (C9:0) and decanoyl-CoA (C10:0)] cannot. However, it is not clear why the *Vibrio* FadR and EcFadR differ greatly in the amplitude of fatty acid sensing. Given that isothermal titration calorimetry (ITC) can give clues to different binding modes of small molecules to a certain protein and the equilibrium dissociation constants (Kds), we adopted the ITC method to monitor palmitoyl-CoA (C16:0) ligand binding to the three *Vibrio* FadR proteins (plus EcFadR) (Figure 4, Figure S4). Comparative analyses of the ITC profiles clearly showed that each EcFadR monomer binds palmitoyl-CoA ligand at the ratio of 1:1 (Figure 4A), which is consistent with the scenario seen with structure of *E*. coli FadR liganded with myristoyl-CoA (**Figures 6A,B**) (van Aalten et al., 2001). In contrast, each monomer of ValFadR bound 2 molecules of palmitoyl-CoA (Figure 4B). As anticipated, the similar ligand-binding pattern was also seen in VcFadR (Figure S4A) and VpFadR (Figure S4B). In addition, the ITC measurements showed that the binding of *Vibrio* FadRs (VcFadR, VpFadR, and ValFadR) palmitoyl-CoA (Figure 4B, Figure S4) are 4- to 10-fold higher than that of EcFadR (Figure 4A) [the value of Kd1 exhibited appreciably lower (3.17-fold for ValFadR (Figure 4B), 7.07-fold for VcFadR (Figure S4A), and 5.06-fold for VpFadR (Figure S4B)]. Obviously, it was consistent with the fact that *Vibrio* FadR is a much more efficient regulator than *E. coli* FadR (Iram and Cronan, 2005). Together, our ITC findings suggested that ligand-binding of *Vibrio* FadR is in a manner distinct from that of EcFadR (Figure 4, Figure S4), which is consistent with the scenario seen in structure of VcFadR reported by Shi *et al*. (Shi et al., 2015).

### Overall architecture of ValFadR

**Figure 5:**
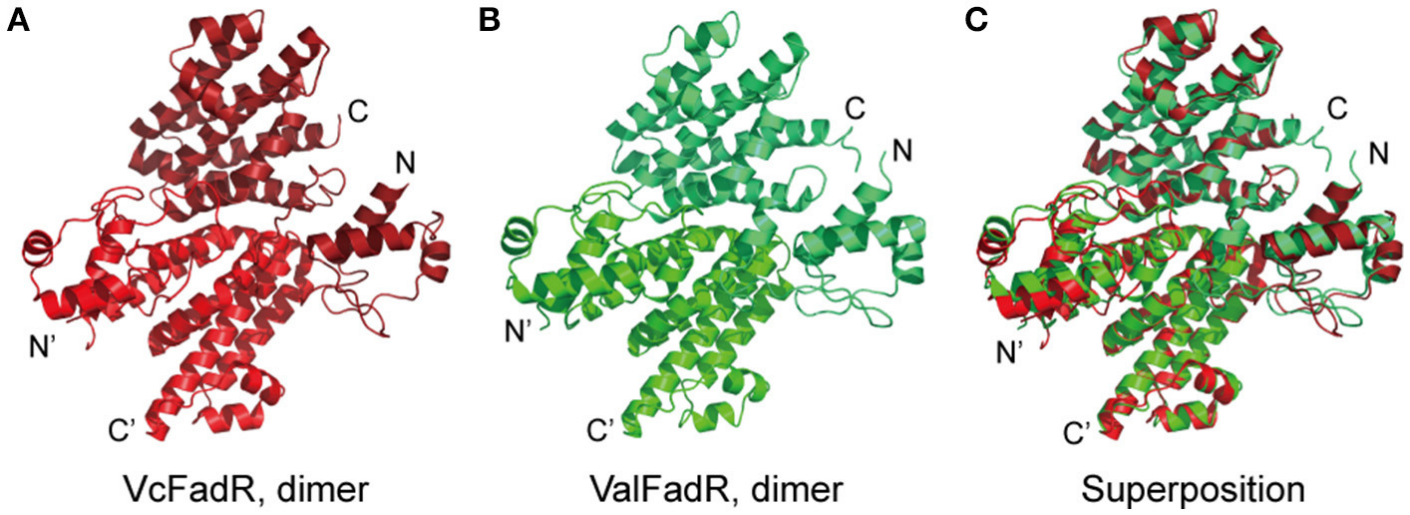
Structural comparison of the FadR proteins of *V. cholerae* and *V. alginolyticus*. **(A)** Ribbon structure of the dimeric FadR protein of *V. cholerae*. **(B)** X-ray structure of the *V. alginolyticus* FadR protein in dimer. **(C)** Structural superposition of the two FadR proteins from *V. alginolyticus* and *V. cholerae*. Vc, *V. cholerae;* Val, *V. alginolyticus*.

In our pilot trials of protein crystal screening, only the ValFadR amongst the three *Vibrio* FadR proteins (VcFadR, VpFadR, and ValFadR) is successful. The structure of apo-ValFadR was determined at 2.35 Å (Table 1). In an agreement with those of our gel filtration (Figure S2), ValFadR forms a dimer and belongs to space group of P4_3_2_1_2, in which each asymmetric unit contains one FadR protomer (Table 1, **Figures 7A,D**). Solution structure of dimer is consistent with the previously-reported EcFadR (van Aalten et al., 2000) and VcFadR (Shi et al., 2015) (Figure 5, Figure S5). The analyses of structural superposition suggested that overall architecture of ValFadR (Figure 5B) can match well that of VcFadR (Figures 5A,C). The protomer is composed of two functional domains, the N-terminal DNA-binding domain, the C-terminal ligand-binding domain and one linker connecting these two domains (Figure S5). The N-terminal DNA-binding domain adopts a typical wHTH fold motif and includes three α helices (α1, α2, α3) and two β strands (β1 and β2) (Figures S1, S5). The C-terminal ligand binding domain forms an α-helix bundle and includes eight helices (Figures S1, S5). Sequence alignment revealed that ValFadR has more 40 residues (138–177 aa) insertion between helices α6 and α7 than EcFadR (Figure S1). The ValFadR structure shows that these 40 residues elongate helices α7, and form two helices, αA and αB, and some linking loops (Figures S1, S5B,E), which is generally similar to that of VcFadR alone (Figure 5) (Shi et al., 2015). Except the insert of 40 residues, the structure of apo-ValFadR (Figures S5B,E) is highly similar to that of apo-EcFadR (Figures S3A,D) with r.m.s.d over 223 Cα atoms (one protomer) of 1.5 Å (Figures S5C,F).

**Table 1:** Statistics on data collection and structure refinement.

	**Native**	**Palmitoyl-CoA complex**
**DATA COLLECTION**		
Diffraction source	Rigaku MicroMax007HF	Rigaku MicroMax007HF
Space group	P4_3_2_1_2	P2_1_
**CELL DIMENSIONS**		
a, b, c(Å)	76.00, 76.00, 144.41	52.78, 88.48, 61.65
α, β, γ(°)	90, 90, 90	90, 93.13, 90
Wavelength(Å)	1.5418	1.5418
Resolution range(Å)^a^	18.47–2.35 (2.48–2.35)	61.55–2.40 (2.53–2.40)
Unique reflection	17,995 (2,322)	20,456 (2,570)
Redundancy	4.8 (4.7)	4.5 (4.1)
*I/σ*	14.0 (3.4)	8.7 (1.9)
Completeness (%)	97 (98.6)	92.7 (84.1)
*R_*merge*_*(%)^b^	7.1 (41.0)	6.9 (39.3)
**STRUCTURE REFINEMENT**		
No. of reflections (working)	16,195	18,435
No. of reflections (test)	1,800	2,021
*R_*cryst*_/R_*fre*_*_e_(%)	21.8/25.6	19.0/24.7
No. of total atoms	2,312	4,675
No. of non-H protein atoms	2,200	4,328
No. of Ni^2+^	1	–
No. of palmitoyl-CoA atoms	–	260
No. of water molecules	111	87
Average B-factors(Å^2^):	72	49
Protein	72	48
Ni^2+^	45	–
Palmitoyl-CoA	–	62
Water	51	41
**RMS DEVIATION FROM IDEAL GEOMETRY**		
Bond length(Å)	0.003	0.002
Bond angle(°)	0.663	0.677
**RAMACHANDRAN PLOT (%)**		
Most favored regions	95.91	95.66
Additional allowed regions	4.09	3.58
Generally allowed regions	0	0
Disfavored regions	0	0.75

a *Values in the parentheses are for the data in the highest resolution shell*.

b *R_merge_ = ∑|I_i_− I_m_|/∑I_i_, where I_i_ is the intensity of the measured reflection, and I_m_ is the mean intensity of all of the symmetry-related reflections*.

### Structure of ValFadR liganded with palmitoyl-CoA

**Figure 6:**
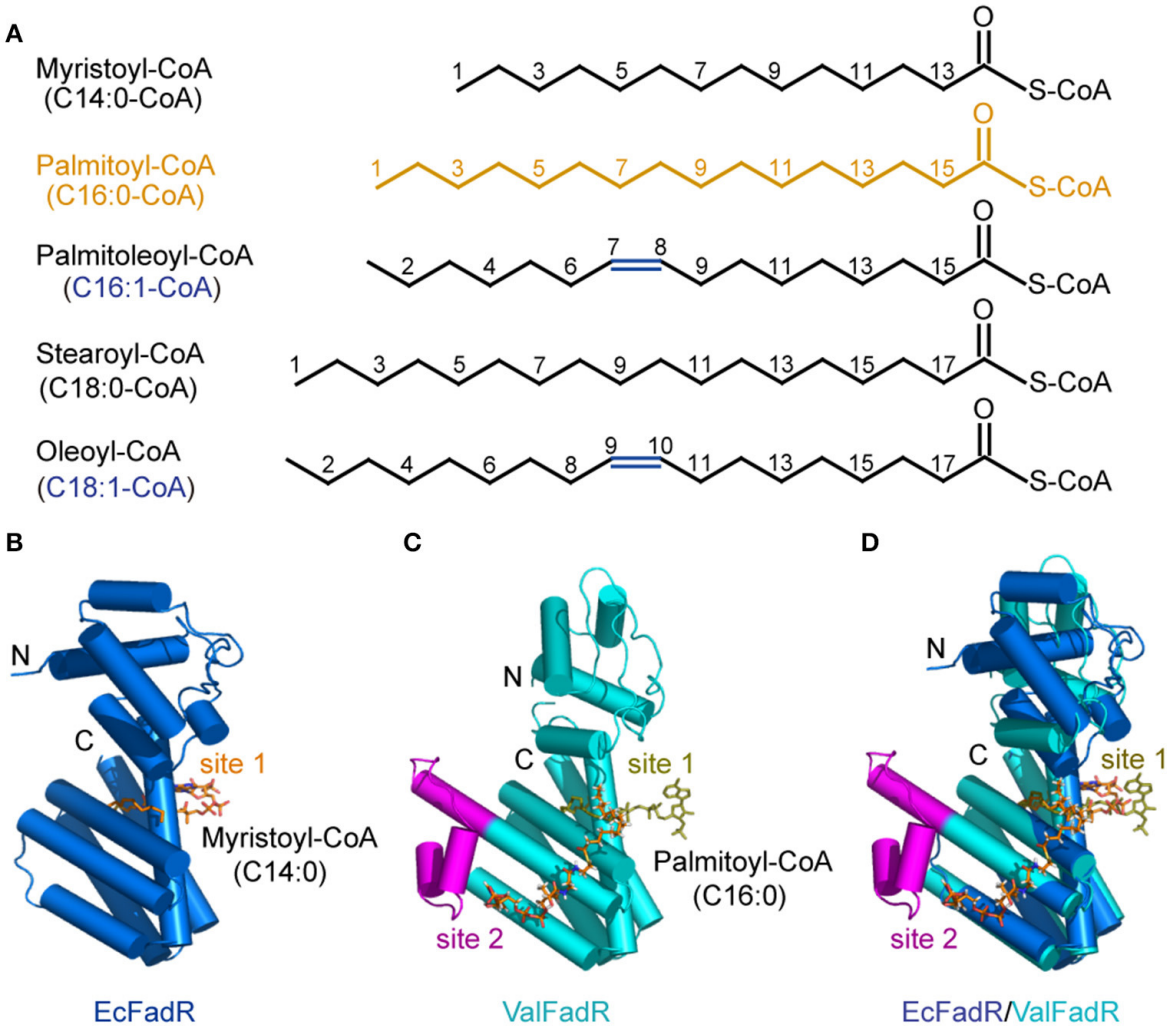
Chemical structures of the representative FadR ligands and structural comparison of the liganded-ValFadR with the EcFadR-ligand complex structure. **(A)** Chemical structures of LC fatty acyl-CoAs, the ligands for FadR regulatory proteins. The palmitoyl-CoA ligand that was successfully crystalized into the FadR protein is highlighted in orange, whereas the double bond is indicated in blue. **(B)** Complex structure of the monomeric form of EcFadR liganded with myristoyl-CoA ligand. The architecture of the EcFadR (PDB: 1H9G) is shown in blue cylinder, whereas the ligand of myristoyl-CoA is given in orange stick. **(C)** Complex structure of the monomeric form of ValFadR liganded with palmitoyl-CoA ligand (PDB: 5DV5). The protomer form of ValFadR is shown in cyan cylinder, and the extra-40aa insert is given in magenta. The ligands are shown in sticks, one of which is of light green in the binding site 1 and the other one present in site 2 is indicated in red. **(D)** Superposition of the monomeric structure of ValFadR-ligand and EcFadR-ligand structure via their C-terminal ligand-binding domains.

**Figure 7:**
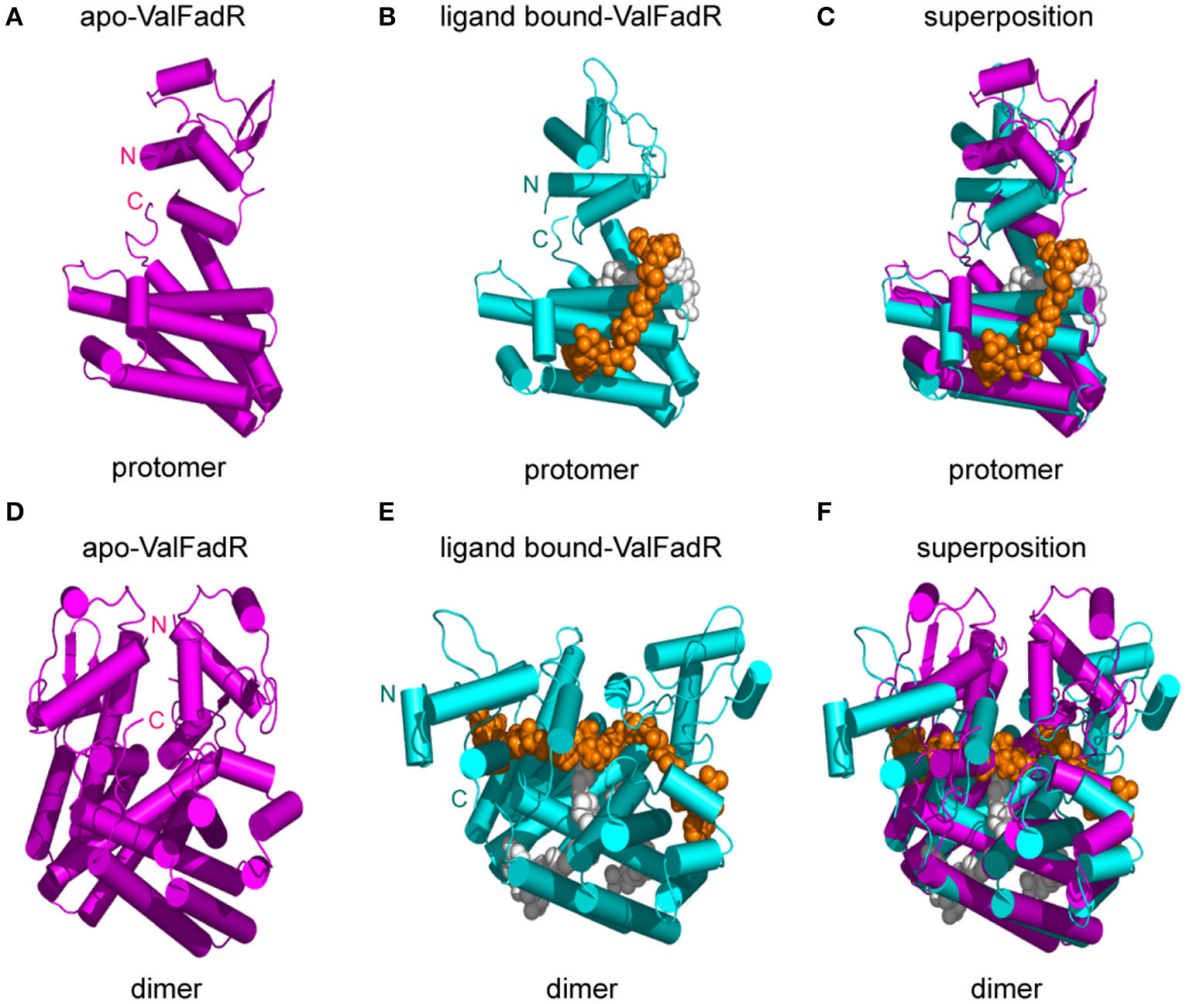
Structural comparison of the apo- and ligand bound-FadR protein from *V. alginolyticus*. Monomeric **(A)** and dimeric **(D)** structures of apo-ValFadR protein are shown in magenta cylinder (PDB: 5XGF). Protomer **(B)** and dimeric **(E)** structures of ligand-bound ValFadR protein are shown in cyan cylinder (PDB: 5DV5). The monomeric **(C)** and dimeric **(F)** structures of the apo- and ligand-bound ValFadR protein are superposed via their C-terminal ligand-binding domains. The ligand in binding site 1 is shown in gray spheres and the other one in binding site 2 is indicated with orange spheres. Val, *V. alginolyticus*; N, N-terminus of the FadR protein; C, C-terminus of the FadR protein.

Unlike the fact that VcFadR was complexed with the ligand oleoyl-CoA (Shi et al., 2015), we obtained the crystal of ValFadR-liganded with palmitoyl-CoA that was diffracted by X-ray to 2.4 Å (Table 1). The asymmetric unit contains one ValFadR dimer and four palmitoyl-CoA molecules, in a 1:2 stoichiometry (Figures 6C, 7B,E) as reported for VcFadR (Shi et al., 2015), whereas EcFadR binds one molecule of ligand (myristoyl-CoA) per monomer (Figures 6A,D). It fully validated the general conclusion from the aforementioned ITC experiments (Figure 4, Figure S4). The two ligand-binding sites in ValFadR are similar to those observed with VcFadR (Figure S6) (Shi et al., 2015). One of the ligand molecules binds to ValFadR in the site that is equivalent to EcFadR ligand-binding site (designated site 1), and the second ligand binds to the pocket derived in part from the 40-residue insertion region. The site 1 is mainly formed by the C-terminal domain (Figures S6A,B), whereas the site 2 is formed by both the N- and C-domains (Figures S6C,D). The electron density of the acyl group in the ligand in site 1 is well defined whereas the phosphorylated adenosine head group is not clear (Figures S6A,B). The phosphorylated adenosine head of the palmitoyl-CoA ligand in one protomer interacts with several residues from the other protomer, including hydrogen bonds between Arg245 and the phosphate group. The pyrophosphate group interacts with residues of both Arg253 and Tyr115 via hydrogen bonds (Figures S6A,B). For the ligand in site 2, the phosphorylated adenosine head group is well defined in electron density while the acyl group lack this ability (Figures S6C,D). The phosphorylated adenosine head group inserts into the pocket derived from the part of the 40-residue insertion region. Among it, the adenosine group stacks with residues Tyr153 and Met187 and forms hydrogen bond with Asn130 and Tyr184. The phosphate group forms hydrogen bond with residue Lys156 and the pyrophosphate forms hydrogen bond with residues Arg126 and Arg191 (Figures S6C,D).

Structural comparison revealed that the C-terminal domain of ValFadR is distinctly varied from that of EcFadR-ligand complex structure (Figure 6D), which is due to the different stoichiometry of ligand binding, with an r.m.s.d larger than 3 Å over 100 Cα atoms. In contrast, the N-terminal domain is similar to that of EcFadR with an r.m.s.d 1.0 Å over 70 Cα atoms (Figure 6D). The major difference between ValFadR and EcFadR lies in the N-domain orientations. The second ligand-binding site involves some residues of the N-terminal domain (Figures S6A,D) and the bound second ligand made the N-domain could not take the similar orientation observed in apo-ValFadR and EcFadR (Figures 6B–D). The large-scale movement of the N-domain leads to distance changes between the two DNA-binding domains of the protein dimer and places the domains in a conformation not suitable for DNA binding. It represents structural basis for ligand-triggered ValFadR-DNA dissociation.

### Conformational alteration of ValFadR by ligand binding

Structure superposition showed that the major difference between apo-ValFadR (Figures 7A,D) and liganded-ValFadR (Figures 7B,E) lies in the varied N-domain orientations (Figures 7C,F). Similar scenario also occurred between ligand-bound ValFadR and ligand-bound EcFadR (Figures 6B–D). To illustrate the mechanism whereby ligand-binding leads to the conformational change, both domains of apo-ValFadR and ligand-bound ValFadR are superposed. The N-domains show similar conformations with r.m.s.d 0.9 Å over 65 Cα atoms, so do the C-domains with r.m.s.d 1.3 Å over 173 Cα atoms (Figures 7C,F). These results suggest that the ligand-binding does not alter the overall conformation of both domains but mainly changes the orientation of N-domain (Figures 7C,F). The ligand in the site 1 does not interact with the N-terminal domain and the linker helix, indicating that this ligand-binding does not contribute to the large-scale domain swing. Also, there is no significant domain movement observed in EcFadR upon the ligand-binding (equivalent to site 1 of ValFadR). The ligand in the site 2 interacts with helix α4 of the linker. Interestingly, this helix α4 exists as a standard helix without a B factor significantly higher than the other local residues and did undergo a conformational change from helix to loop upon ligand binding (Figures S6C,D), which is quite different from that of VcFadR (Shi et al., 2015). The second ligand wedged into the interface of N- and C- domain via its acyl chain tail and alters the orientation of helix α4 as well as the N-domain, which is proposed to be a major reason for the large-scale domain swing upon binding the second ligand. Also, the additional ligand in one protomer interacts with many residues from the other protomer as described above, which definitely affects the dimeric interface and causes slightly relative movements between two protomers.

### Physiological roles of *Vibrio* FadR and the additional ligand-binding site 2

Genetic amenability in *V. cholerae* allowed us to create the Δ*fadR* deletion mutant, using the method of homologous recombination (Figure S7A). Following PCR-based identification, we harvested the interested Δ*fadR* mutant (Figure S7B). Given the fact that the ligand-binding site 2 of the *Vibrio* FadRs is mainly involved in the insertion of 40-residues (138-177aa, Figure S1, Figure 5), we therefore designed a site 2-deficient *fadR* mutant (Δ42 without 136-177aa) and engineered it into the Δ*fadR* mutant of *V. cholerae*. Functional complementation with the wild-type and/or Δ42 mutant of *fadR* gene into the Δ*fadR* to probe the *in vivo* role of *fadR* and the ligand-binding site 2 in the context of lipid metabolism in *V. cholerae*.

**Figure 8:**
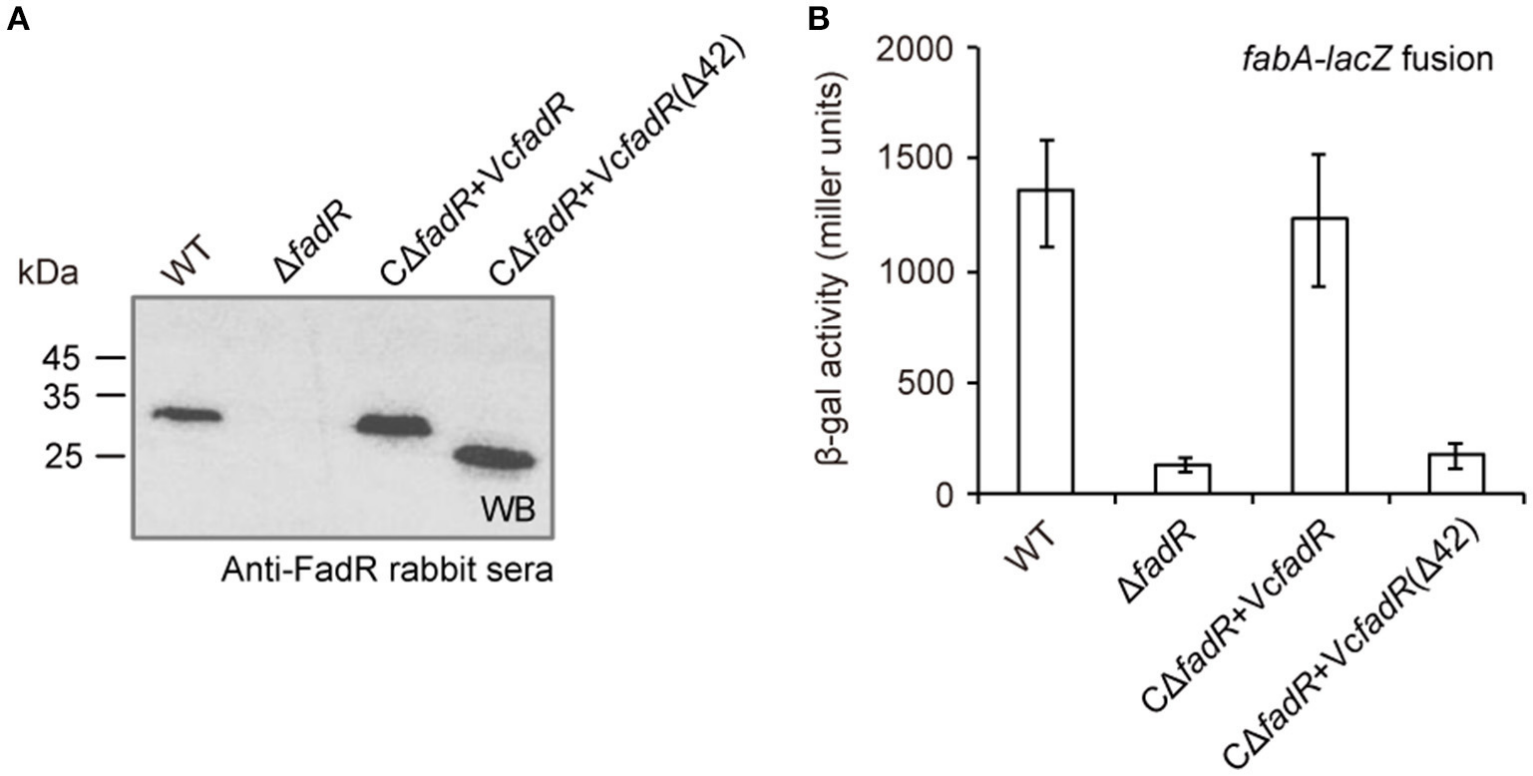
FadR activates expression of *fabA*, an important gene for unsaturated fatty acid synthesis in *V. cholerae*. **(A)** Western blot analyses for *fadR* expression and its derivatives. The primary antibody used here is rabbit sera against *V. cholerae* FadR. **(B)** The beta-gal activity of *fabA-lacZ* transcription fusion is significantly decreased in the Δ*fadR* mutant, and can be restored by the functional complementation of the wild-type *fadR* [but not Vc*fadR*(Δ42)]. The *fadR* deletion mutant strain referred to Δ*fadR*, and the two types of complemented strain of Δ*fadR* used corresponded to CΔ*fadR* + Vc*fadR* and CΔ*fadR* + Vc*fadR*(Δ42), respectively. The Vc*fadR*(Δ42) denoted an engineered version of *V. cholerae fadR* with a 42-residue deletion covering the site 2 for ligand binding. Log-phase cultures were collected for assaying the LacZ activity. The β-gal activity is expressed as the mean ± standard deviation and is collected from no less than three independent experiments.

**Figure 9:**
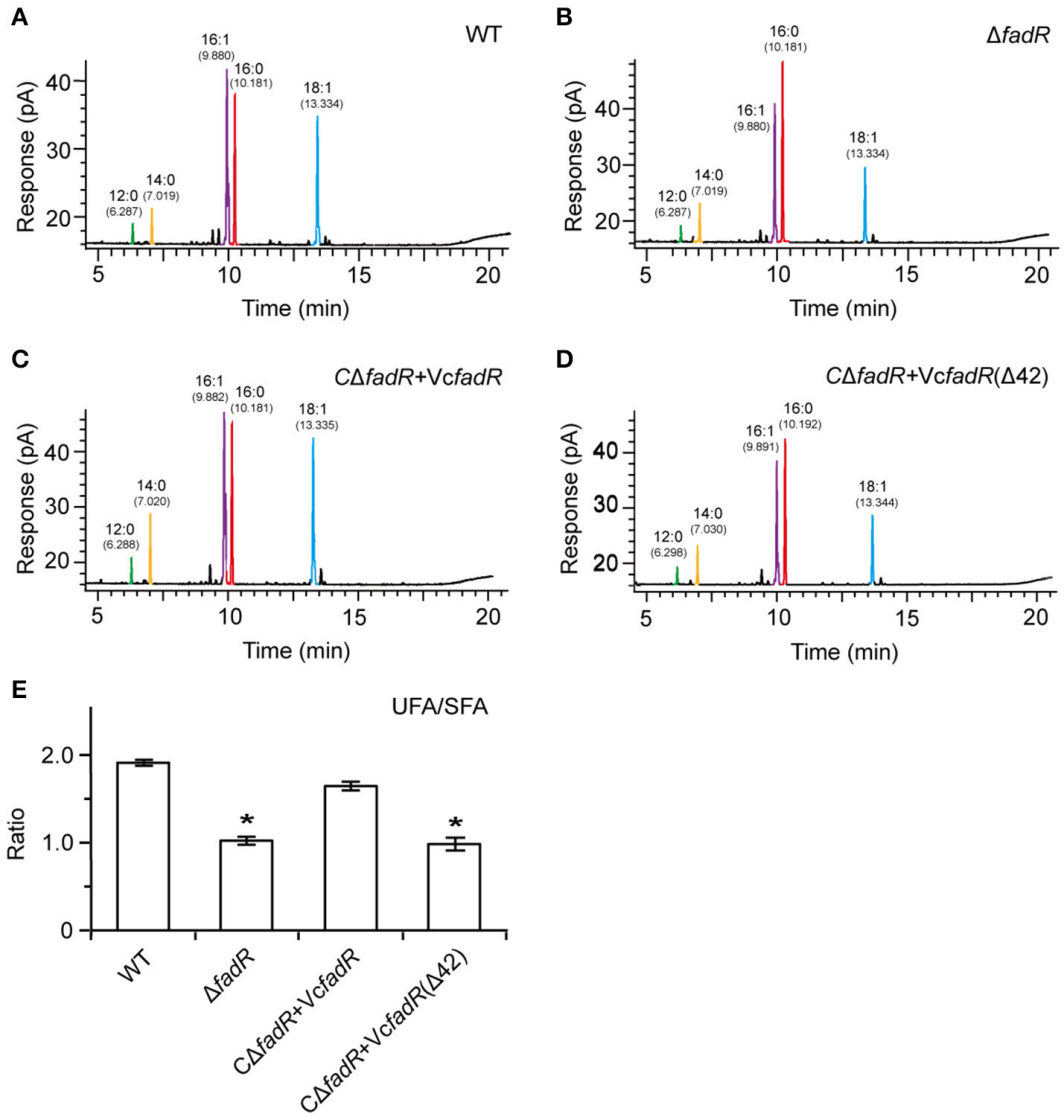
Systematic analyses of fatty acid profile suggests that functional alteration of the *Vibrio* FadR remodels structure of bacterial membrane phospholipids. **(A)** Analyses for the fatty acid composition isolated from the wild-type strain of *V. cholerae*. **(B)** Alteration in the fatty acid compositions in the Δ*fadR* mutant of *V. cholerae*. **(C)** The fatty acid profile in the complemented strain of Δ*fadR* mutant with plasmid-borne *V. cholerae fadR*. **(D)** Failure to recover the fatty acid composition of the *V. cholerae* Δ*fadR* strain using plasmid-driven expression of the *V. cholerae fadR* derivatives that lack the second ligand-binding site [designated as Vc*fadR*(Δ42)]. **(E)** Effects on UFS/SFA ratio of membrane phospholipids by FadR regulatory protein in *V. cholerae*. ^*^Student *t*-test *p* < 0.01.

First, western blot analyses with anti-VcFadR rabbit sera as primary antibody showed that the wild-type of *fadR* gene and its derivative [i.e., the *fadR* (Δ42) mutant] can express well in *V. cholerae*, whereas not in the Δ*fadR* mutant strain of *V. cholerae* (Figure 8A). Subsequently, LacZ-based transcriptional assays revealed that *fabA* expression is decreased dramatically in the Δ*fadR* mutant of *V. cholerae*, and can be enhanced greatly upon over-expression of the wild-type *V. cholerae fadR* (Figure 8B). This result confirmed that FadR acts as an activator for the *fabA* gene of UFA synthesis in *Vibrio*. Of note, the *fadR* (Δ42, lacking the site 2) mutant gene failed to restore *fabA* transcription (Figure 8B), implying the site 2-containing region is critical for VcFadR function. Further analyses of fatty acids composition from *Vibrio* membrane phospholipids showed that (i) UFA/SFA ratio in the Δ*fadR* mutant (Figures 9B,E) is relatively lower than that of the wild-type strain (Figures 9A,E), (ii) this ratio can be restored in the complementary strain (CΔ*fadR*+Vc*fadR*) (Figures 9C,E); and (iii) the *fadR* (Δ42) mutant gene without the site 2 cannot (Figures 8D,E). Therefore, we believed that the *Vibrio* FadR does control/remodel membrane integrity determined by the ratio of UFA to SFA incorporated into bacterial membrane phospholipids (Figure 9) via its positive regulation of the *fabA* transcription (Figure 8).

### Roles of *Vibrio* FadR and the ligand-binding site 2 in bacterial virulence

**Figure 10:**
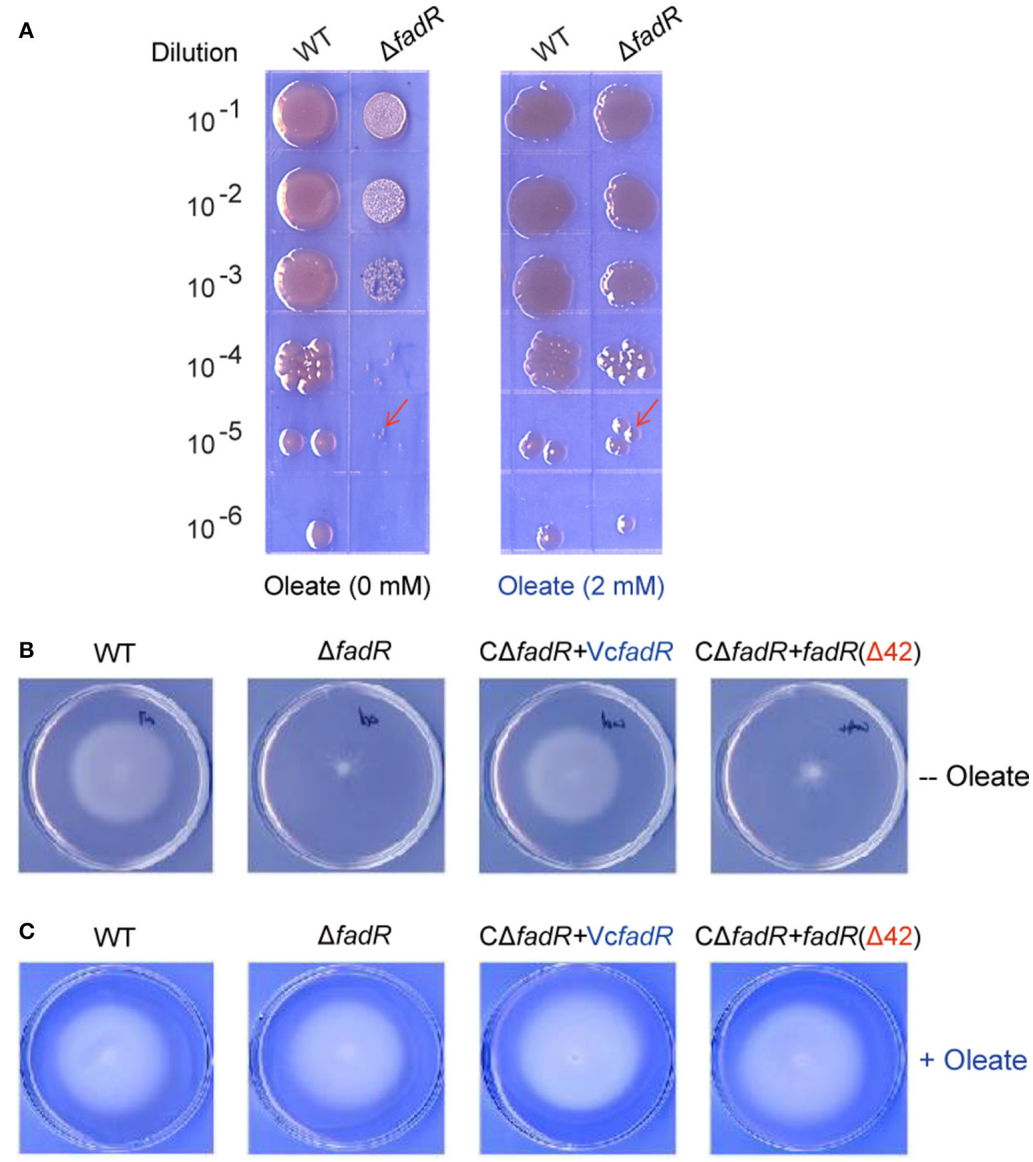
Oleate can rescue the defection of bacterial growth and swarming caused by the inactivation of FadR. **(A)** The Δ*fadR* mutant exhibited the phenotype of small colony size, and growth defect was restored by an addition of oleate into LB media. The two *V. cholerae* strains used here included WT (C6706) and FYJ639 (Δ*fadR*). Bacterial cultures in serial 10-fold dilutions were spotted (from *top* to *bottom*) on plates of LBA solid medium, and kept at 37°C for 18 h. On the LBA plate without any addition of oleate (Left panel), the single colony size of Δ*fadR* is much smaller than that of the wild-type. However, the colonial size of the two strains is comparable upon the supplementation of oleate (Right panel) into the LBA plates. The typical colonies are indicated with red arrows. A representative result is given. **(B)** The ability of bacterial swarming, a virulence-associated trait, was impaired by the deletion of VcFadR, but rescued by the functional complementation of the wild-type *fadR* (but not *fadR*(Δ42)) and *in vitro* supplementation of oleate can rescue **(C)**. For the swarming assays, the log-phase culture appropriately diluted (~10 ul) is spotted on semi-solid 0.3% agar LB plates and kept overnight at 37°C.

Similar to the phenotype of the *fadR* insertional mutant strain of *V. vulnificus* (Brown and Gulig, 2008), the *fadR* deletion mutant strain of *V. cholerae* also displays slower growth in LB liquid media and colony of smaller size on LBA plates than the wild-type strain (Figure S8, Figure 9A). The growth/morphological defects were significantly restored by both plasmid-borne expression of the wild-type VcFadR (Figure S8) and the addition of exogeneous oleate (Figure 10A). However, the expression of *fadR* (Δ42) with ligand-binding site 2 deleted (Figure 8A) failed to complement the phenotype of defective growth (Figure S8). In light that *fadR* (Δ42) mutant neither augments the decreased expression of *fabA*, an important gene for UFA synthesis (Figure 8B), nor rescues the dysfunction in fatty acid composition of phospholipids in the Δ*fadR* mutant strain of *V. cholerae* (Figures 9D,E), we favored to hypothesize that the activation of fatty acids synthesis by FadR has significant impacts on bacterial growth and the ligand-binding site 2 is critical for FadR function *in vivo*.

**Figure 11:**
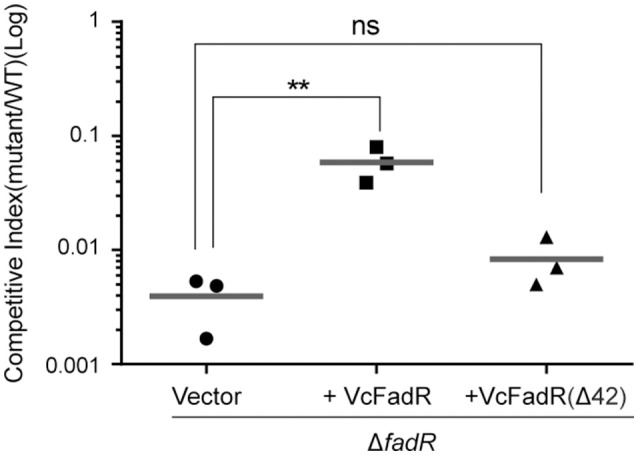
Roles of *V. cholerae* FadR and the new ligand-binding site 2 in bacterial virulence. Effects exerted by *V. cholerae* FadR on colonization in the infant mouse model. Δ*fadR* mutants containing vector control, Vc*fadR* and Vc*fadR* (Δ42) were co-inoculated into infant mice with wild-type at 1:1 ratio. After 18-h colonization, the colonized bacteria were enumerated on selective agar plates. Competitive index is calculated as output ratio of mutant to wild-type normalized against input ratio of mutant to wild-type. Horizontal line: mean of CI. ^**^Student *t*-test *p* < 0.01; ns, no significance.

Given that the opportunistic pathogen *V. vulnificus* FadR is necessary for bacterial infection in mice (Brown and Gulig, 2008), it is of much interest to test if site 2 has a role in virulence-associated traits as well as infection competency in *V. cholerae* (Figures 10, 11). Thus, we examined the possible relevance of *fadR* (and/or the ligand-binding site 2) to bacterial swarming, a virulence-associated trait. As expected, the swarming ability was impaired greatly upon the removal of *fadR* from *V. cholerae* (Figure 10B) (Brown and Gulig, 2008). Functional complementation with VcFadR recovered it to that of the parental strain (Figure 10B). Although the expression of VcFadR (Δ42) (Figure 8A) failed to complement the loss of swarming ability, supplementation of exogenous oleate rendered them indistinguishable in the swarming assays (Figure 10C). We therefore anticipated that dysfunction of swarming/mobility is related to altered surface architecture of membrane phospholipids. Subsequently, we performed infant mouse colonization competition assays (Gardel and Mekalanos, 1996). To rule out the possible effects of delayed growth of Δ*fadR* mutant, we subjected them to the *in vitro* colonization trials on LBA plates supplemented with oleate prior to colonization experiments of mice *in vivo*. The result showed that the delayed growth phenotype of the Δ*fadR* mutant does not affect greatly the *in vitro* output [i.e., the ratio of colony numbers (WT/Δ*fadR*)] (Figure S10). Thus, we compared the colonization abilities of the wild-type strain with the Δ*fadR* mutant strain and the complementary strain of *fadR* mutant (with either wild-type *fadR* or *fadR* [Δ42, lacking site 2]). We found that the Δ*fadR* mutant is profoundly defective in colonization (the competitive index is around 0.004) (Figure 11), suggesting that FadR plays a critical role in *V. cholerae* virulence. Complementation with the wild-type *fadR* partially restored colonization of Δ*fadR*, whereas the plasmid containing *fadR* (Δ42) failed to recover the Δ*fadR* colonization (Figure 11). It seems possible that the site 2-containing region (42-residues long, 136-177aa) is critical for *V. cholerae* FadR as a functional virulence factor. In fact, we also noted expression of the two major virulence factors *ctxA* (Figure S9A) and *ctpA* (Figure S9B) are slightly inhibited by inactivation of *fadR*. This control of virulence factors was assigned by Kovacikova et al. (2017) to the indirect modulation of ToxT by FadR via two different mechanisms. Hence, our findings might be additional to the linker of bacterial fatty acid metabolism and *Vibrio* pathogenesis.

## Discussion

Different organisms have developed diversified mechanisms to tightly modulate fatty acid synthesis as fatty acids are energetically-expensive molecules to produce. To date, six bacterial transcription regulators have been identified. Three of them [FapR (Schujman et al., 2003), DesR (Aguilar et al., 2001; Zhu et al., 2006), and FabT (Lu and Rock, 2006)] are found in Gram-positive bacteria, while the other three members [FadR (Henry and Cronan, 1991; Cronan and Subrahmanyam, 1998; Campbell and Cronan, 2001), FabR (McCue et al., 2001; Zhang et al., 2002), and DesT (Zhang et al., 2007)] are derived from Gram-negative bacteria. Unlike the prototypical version of FadR regulatory protein, the *E. coli fadR* protein product (van Aalten et al., 2000, 2001; Xu et al., 2001), the description of three *Vibrio* FadR homologs (ValFadR, VpFadR, and VcFadR) from our groups and recent report by Shi and coworkers (Shi et al., 2015) defined a unique mechanism for bacterial fatty acid sensing in the genus of *Vibrio* (Figure 1). Although the *Vibrio* FadR proteins were derived from different species (*V. alginolyticus* from our group and *V. cholerae* for Kull's group), the structures we obtained are almost identical, both of which underlined that the insert of 40-residues constitute an extra-domain rich in α-helix (Figures S1, S5, Figure 5). Further dissection for two types of *Vibrio* FadR-ligand complexes [ValFadR/Palmitoyl-CoA (C16:0) from our group plus VcFadR/Oleoyl-CoA (C18:1) from F. Jon Kull's group] consistently reveal that the so-called “40-aa insert” of *Vibrio* FadR regulatory protein constitutes the second ligand-binding site in that it already retained the ligand-binding site 1 that is highly similar to that of *E. coli* FadR (Figure 6, Figure S6). This scenario seen with *Vibrio* FadR regulator sounds unusual, but not without precedent in that (i) the cAMP-receptor protein (CRP) requires binding of two cAMP molecules each monomer for its function (Passner and Steitz, 1997); (ii) BenM, a LysR-like regulator, has two ligand-binding sites for effector benzoate (Ezezika et al., 2007). Subsequently, the question raised is what could be a physiological explanation for this unique mechanism. We speculated that unlike the paradigm enteric bacterium *E. coli* colonizing the digestive tract rich in fatty acids, *Vibrio* species residing in aquatic environments with low level of fatty acids precipitated on the sediment (Giles et al., 2011) have to evolve a more efficient system for fatty acid uptake, rendering its fatty acid-responsive FadR regulator to select an additional ligand-binding site.

Indeed, complex structures of two types of the ligand/*Vibrio* FadR from our group (Figure 4, Figure S5) and report by Shi et al. (2015) allow us to better understand the structural/molecular basis for crosstalk/response of *Vibrio* FadR to effectors. As illustrated in Figure 6 and Figure S6, the head of CoA for the second ligand is well fixed and recognized, and the tail of fatty acid chain is inserted between N-terminus and C-terminus of ValFadR and a two-body interface. That is why we hypothesize that fatty acyl-CoA of different lengths might synergistically interfere with *Vibrio* FadR. In the dimeric form of *Vibrio* FadR protein, the ends of the four lines of fatty acyl chains converge at the same point, which determine the inability of the limited space to hold fatty acyl chains with much longer length. Probably, the acyl chain of both C16 in ValFadR (Figure 6, Figure S6), and C18 in VcFadR (Shi et al., 2015) seemed to be quite close to the its length limit. The fact that extensive ligand-triggered conformation changes seen with two FadR proteins (ValFadR and VcFadR, Shi et al., 2015) more completely impaired the DNA binding than that of *E. coli* FadR protein, provided structural explanation for our former observation that addition of oleate can result in almost full de-repression of *Vibrio fad* regulon, like *fadBA* (Iram and Cronan, 2005). Not only have we elucidated the contribution of the extra 40-aa insert to the function of *Vibrio* FadR protein in the context of lipid metabolism, but also discovered its relevance to bacterial infections (Figures 10, 11). Additionally, the uniqueness of this insert in*Vibrio* might determine that the fatty acid sensing by FadR with double ligand-binding sites is exclusively distributed into the phylum of *Vibrionaceae* from ɤ-proteobacteria.

Together with the recent Kavocikova's discovery that virulence control by *V. cholerae* FadR is due to indirect cross-talking with ToxT, a master virulence regulator, via two independent mechanisms (Kovacikova et al., 2017), our results constituted additional input to better understanding of link between fatty acid metabolism and bacterial pathogenicity, i.e., membrane integrity of phospholipids is crucial for bacterial competitive colonization. The structural characterization of ValFadR regulator defines an additional example in the context of the diversified regulation of bacterial fatty acid metabolism (Marrakchi et al., 2002; Zhang and Rock, 2009). We hypothesized that the *Vibrio* FadR regulator has evolved this additional ligand-binding site to gain an advantage of infection competency in bacterial-host battle. Given the significant contribution of FadR regulatory protein to pathogenesis of certain *Vibrio* species, it is possible that development of small molecule drugs/inhibitors targeting the FadR-mediated mechanism for bacterial fatty acid sensing might be efficient therapeutics against severe infections by the zoonotic *Vibrio* pathogens.

## Materials and methods

### Ethics statements

Animal care and treatment were conducted in accordance with institutional guidelines and the protocols at Zhejiang University, China. Five-day-old CD1 mice were housed in a temperature-controlled room with proper darkness-light cycles, fed with a regular diet, and maintained under the Experimental Animal Center of Zhejiang University, China. The mouse experimental design and protocols used here were approved by the Ethics Committee of Zhejiang University, China (ZJU20170800).

### Bacterial strains and growth conditions

*E. coli* strains used here are derivatives of the wild-type K-12 strain (Table S1). *Vibrio* strains used here included *V. cholerae* EI Tor C6706 (Joelsson et al., 2006), *V. parahaemolyticus* ATCC 17802 and *V. alginolyticus*. *E. coli* SM10 (λ-*pir*) was used as the donor strain for conjugations with *V. cholerae*. These strains were routinely maintained in LB medium (Luria-Bertani medium containing 10 g of tryptone, 5 g of yeast extract and 10 g of NaCl per liter) or on LB agar plates (Feng and Cronan, 2011). LB without NaCl and containing 6% (wt/vol) sucrose was used for a selection of the strain with homologous recombination. The growth temperatures used are either 30° or 37°C. When necessary, antibiotics were added as follows (in μg/ml): tetracycline, 20 for *E. coli* and 2 for *V. cholerae*; ampicillin, 100; kanamycin, 50; and streptomycin, 100 (Feng and Cronan, 2009a).

### Plasmids and DNA manipulations

*V. cholerae fadR* gene was previously cloned pET16b vector (Iram and Cronan, 2005). The two *fadR* genes from *V. parahaemolyticus* and *V. alginolyticus* were separately amplified with PCR. They were directionally cloned into the pET28 expression vector. The resultant recombinant plasmids were introduced into *E. coli* BL21 (DE3) for large-scale production of the FadR protein. As we previously described in the synthesis of the *Brucella bioR* gene *in vitro* with little change (Feng et al., 2013), overlapping PCR was used to create a mutant *fadR* gene (*fadR*(Δ42)) with the deletion of 42 residues (136-177aa). The pWM91 vector is applied for construction of *fadR* knock-out plasmid (Metcalf et al., 1996). The expression plasmid pBBR1-MCS3 is used for functional assays of the *fadR* gene in different versions (Vc*fadR* and the Vc*fadR* mutant Δ42), Table S1) in *V. cholerae* (Kovach et al., 1995). All plasmid constructs were validated by PCR detection (Table S2) and direct DNA sequencing (Zhang et al., 2014).

### In-frame deletion of *V. cholerae fadR* and genetic complementation

*V. cholerae* El Tor C6706 (Joelsson et al., 2006) was used as the wild-type strain in this study unless otherwise noted. In-frame deletions were constructed by cloning of the upstream (and downstream) regions neighboring the target *fadR* gene (*vc1900*) into the suicide vector pWM91 containing a *sacB* counter-selectable maker (Metcalf et al., 1996). The upstream (and/or downstream) DNA fragments (of 700 bp long) flanking *fadR* were amplified by PCR using the two pairs of specific primers (L-flank *fadR*-5′/L-flank *fadR*-3′ and R-flank *fadR*-5′/R-flank *fadR*-3′) with the high-fidelity Prime STAR DNA Polymerase (Table S2). The flanking DNA sequences in the upstream (and downstream) of the *fadR* were overlapped and cloned into the two restriction sites of pWM91 (SacI and BamHI), giving knockout plasmid pWM91-*fadR*_KO_. Then the plasmid was introduced into the *V. cholerae* strain by conjugation from SM10 (λ-*pir*) and a single crossover event was selected by resistance to streptomycin and ampicillin. The selection growth in the absence of ampicillin and streptomycin assures that double crossover events occur. Finally, the Δ*fadR* mutant was given via sucrose-based screening. The proper genetic exchange event was confirmed by multiplex-PCR and direct DNA sequencing.

For functional complementation, the wild-type *fadR* gene of *V. cholerae* plus its Vc*fadR* mutant (*fadR*(Δ42)) were directionally inserted into the pBBR1-MCS3 vector via the two sites (KpnI and XbaI), giving plasmids like pBBR1-MCS3-*fadR* (Table S1). The resultant plasmids were transformed into *E. coli* SM10 (λ*-pir*), and then conjugated into the *V. cholerae* Δ*fadR* mutant. Tran-conjugants were selected on LB plates containing streptomycin and tetracycline. All the constructs were verified by direct DNA sequencing of PCR products.

### Anti-FadR rabbit sera and western blot

Anti-sera against VcFadR were prepared as previously described (Shao et al., 2011). Pre-immune sera were collected from New Zealand White rabbits (female, 1.5 kg) and then animals were injected subcutaneously at multiple sites with approximately 1 mg/kg of purified FadR recombinant protein emulsified with Freund's complete adjuvant (1:1). After 2 weeks, each rabbit received the first booster injection with the same antigen concentration emulsified with Freund's incomplete adjuvant (1:1). One week later, each rabbit received the second booster injection with the same antigen concentration emulsified with Freund's incomplete adjuvant (1:1). Then, anti-serum samples were collected when the booster injection was administered 7 days later. The sensitivity and specificity of antibody were evaluated routinely by ELISA and Western blot (Feng and Cronan, 2009b). Using the rabbit sera against VcFadR protein as primary antibody, Western blotting analyses were conducted to detect expression of *fadR* gene (and its derivatives) in the wild-type (or Δ*fadR* mutant) strain of *V. cholerae*.

### Growth phenotypes and swarming assays

Growth phenotypes of *V. cholerae* and its derivatives were examined using both LBA solid plates and measurement of growth curves. To determine effect of colony size by the deletion of *fadR*, bacterial cultures in series of 10-fold dilution were spotted on solid LBA plates and maintained overnight at 37°C. Similarly, the cultivation of *V. cholerae* species in liquid LB media at 37°C was monitored for plotting the growth curves. For swarming assays, log-phase cultures were diluted appropriately (10 μl each) is spotted on semi-solid 0.3% agar LBA plates and kept overnight at 37°C prior to visualization of mobility. Over three independent trials were conducted.

### Assays for competition colonization in infant mice

Following the protocol established by Davies et al. (2011) with minor modifications, we evaluated the role of *fadR* in bacterial virulence through the competitive colonization experiments in the model of infant mouse. Briefly, the wild-type strain of *V. cholerae* (either C6706 or C6706 *lac*^−^) and its mutants were cultivated on LBA plates with streptomycin (100 μg/ml) overnight at 37°C. The wild-type and mutant strains were mixed together in LB medium. 100 μl of competition mixtures (about 1 X 10^5^ CFU) were inoculated into a 5-day-old CD1 infant mouse. Serial dilutions of the competition mixture were plated on LBA plates with streptomycin (100 μg/ml) as the selective pressure and enumerated to determine the input ratio of wild-type to the mutant strain. After incubation at 30°C for 18 h, the infant mice were sacrificed, and small intestines were removed and homogenized in 5 ml of LB. Serial dilutions were plated on LBA plates containing streptomycin (100 μg/ml) and enumerated to determine the output ratio of wild-type to the appropriate mutant strain. The competitive index for each mutant is defined as the input ratio of mutant/wild-type strain divided by the output ratio of mutant/wild-type strain.

### Protein expression and purification

Since all the other three FadR proteins of *Vibrio* origins were tagged with hexa-histidine on the N-terminus, similar to the paradigm *E. coli* FadR protein stocked in our lab. The recombinant proteins were routinely prepared as we described before (Zhang et al., 2014). Briefly, when the optical density at wavelength of 600 nm (OD_600_) reached 0.6, the bacterial culture was induced with 0.2 mM isopropyl-β-D-1-thiogalactopyranoside (IPTG) at 30°C overnight. Following lysis by sonication, the cell lysate was clarified by centrifugation and loaded onto a nickel-ion affinity column (Qiagen). After removal of contaminant proteins with washing buffer containing 50 mM imidazole, the 6xHis-tagged FadR protein was eluted in elution buffer containing 150 mM imidazole, concentrated by ultra-filtration (30-kDa cutoff) and exchanged into 20 mM Tris-HCl (pH 7.5) containing 100 mM NaCl. The soluble fractions were further purified by gel filtration with a Superdex 200 10/300 column (GL, GE Healthcare) and the resultant FadR proteins were judged with 12% SDS-PAGE. Protein concentrations were measured using the BCA method with BSA as internal reference (Thermo Scientific).

### Electrophoretic mobility shift assays

As we described earlier with minor modifications (Zhang et al., 2014), electrophoretic mobility shift assays (EMSA) were conducted to test the binding of FadR protein to the DNA targets. In this case, FadR protein of *E. coli* and *Vibrio* were studied. The FadR-specific DNA probes is *fabA* probe which is specific to the fatty acid biosynthesis *fabA* gene. The above double-stranded DNA probe was generated by annealing the two complementary primers in TEN buffer (10 mM Tris-HCl, 1 mM EDTA, 100 mM NaCl; pH 8.0). Generally, the DNA probe was mixed with/without FadR protein in binding buffer and incubated at room temperature. The DNA-protein complexes were separated with native PAGE gels (Feng and Cronan, 2011, 2012; Tang et al., 2014).

### Surface plasmon resonance

To further address the interplay between FadR proteins and the DNA target (such as *fabA*), surface plasmon resonance (SPR) was employed using a Biacore3000 instruments (GE Healthcare) at 25°C. For immobilization on an SA sensor chip, the biotinylated DNA probe of *fabA* was specifically captured by streptavidin on the surface. All the SPR experiments were run in the running buffer (20 mM Tris-HCl, pH7.5, 200 mM NaCl and 0.005% Tween 20) at the flow rate of 30 μl/min. A series of dilutions of protein samples were injected and passed over the chip surface for 2 min. The dissociation phase was followed for 3 min in the same buffer, and the surface was then regenerated with 0.025% SDS for 24 s. Kinetic parameters were analyzed using a global data analysis program (BIA evaluation software), and final graphs were further processed with Origin software.

### Isothermal titration calorimetry

To study the binding of FadR protein to its effector molecule like palmitoyl-CoA, isothermal titration calorimetry (ITC) was conducted using a MicroCal iTC200 (Malvern) with a cell volume of 200 μl. Both FadR samples and the ligand of palmitoyl-CoA were dialyzed against the same buffer (20 mM Tris-HCl pH7.5, 100 mM NaCl) to minimize artifacts due to minor differences of the buffer composition. Prior to titration, the sample of FadR protein was injected into the cell, and the syringe was filled with palmitoyl-CoA. All the ITC experiments were performed at 25°C with a reference power of 8 and a stirring speed of 1,000 rpm. The final heat was corrected by subtracting the dilution heat from palmitoyl-CoA titrated to buffer. The raw data was analyzed with the Origin software (note: one set of sites for EcFadR, two sets of sites for VpFadR and ValFadR, and sequential binding sites for VcFadR).

### β-galactosidase assays

To measure bacterial β-gal activity, log-phase cultures of *V. cholerae* carrying the plasmid-borne pTL61T-P*fabA-lacZ* transcription fusion grown in LB media (Table S1) were collected by spinning and suspended in Z-buffer (Gao et al., 2016). The data was obtained from three independent assays.

### Protein crystallization and data collection

All the apo-FadR homologs of three *Vibrio* species (ValFadR, VpFadR, and VcFadR) were subjected to initial crystallization trials at protein level of 10 mg/ml, using hanging-drop vapor diffusion method at 16°C. 1μl of protein solution was mixed equally with the well solution. Consequently, crystals of apo-ValFadR were observed on the condition containing 200 mM Calcium Acetate, 100 mM Sodium Cacodylate pH 6.5 and 7% PEG8000. After 2–3 days, crystals grew to dimension of 0.1 mm by 0.15 mm by 0.1 mm. To obtain the crystal of ValFadR complex with ligand, a 2-fold molar excess of palmitoyl-CoA was added to 10 mg/ml ValFadR solution, and incubated for 2 h at 16°C before crystallization. The crystals were grown on the condition (4% Tacsimate, pH 5.0, 11% PEG3350) in 2 weeks. Crystal was transferred to cryo-protecting solution of mother liquor containing 15% glycerol for a few seconds and then quickly flash-cooled in liquid nitrogen and the dataset was collected on a Rigaku R-Axis IV++ image plate using Cu Kα radiation (λ = 1.5418 Å) at 100 K.

### Structure determination and refinement

Diffraction data of protein crystals was integrated and processed using MOSFLM and SCALA from the CCP4 program suite (Collaborative Computational Project, 1994). The structure of ValFadR was determined by molecular replacement using EcFadR structure (PDB: 1E2X) as a search model. The model was first built automatically using PHENIX package (Adams et al., 2002) and then manually with COOT (Emsley and Cowtan, 2004) and structure refinement was performed with Phenix. For the structure of ValFadR and palmitoyl-CoA complex, the apo-ValFadR structure was used as a search model and then was built manually with Coot and refined with Phenix. The stereo-chemical quality of the refined structures was verified with PROCHECK (Morris et al., 1992). All structural representations were prepared with PyMol (DeLano Scientific). The two PDB accession numbers are listed as follows: 5XGF for ValFadR alone and 5DV5 for ligand-bound ValFadR protein.

## Author contributions

YF designed this project; YF, YL, RG, XX, JL, and DL performed experiments and analyzed the data; LB, JZ, HW, BH, and SW contributed reagents and tools; YF, DL, YL, JZ, and SW wrote this manuscript.

### Conflict of interest statement

The authors declare that the research was conducted in the absence of any commercial or financial relationships that could be construed as a potential conflict of interest.
